# Carbon Dots as Sensing Layer for Printed Humidity and Temperature Sensors

**DOI:** 10.3390/nano10122446

**Published:** 2020-12-07

**Authors:** Almudena Rivadeneyra, José F. Salmeron, Fabio Murru, Alejandro Lapresta-Fernández, Noel Rodríguez, Luis Fermín Capitan-Vallvey, Diego P. Morales, Alfonso Salinas-Castillo

**Affiliations:** 1 Department of Electronics and Computer Technology, University of Granada, 18010 Granada, Spain; jfsalmeron@ugr.es (J.F.S.); noel@ugr.es (N.R.); diegopm@ugr.es (D.P.M.); 2 Department of Analytical Chemistry, University of Granada, 18010 Granada, Spain; fmurru@correo.ugr.es (F.M.); lapresta@ugr.es (A.L.-F.); lcapitan@ugr.es (L.F.C.-V.); alfonsos@ugr.es (A.S.-C.)

**Keywords:** capacitive sensors, moisture content, nanomaterials, screen printing, printed electronics

## Abstract

This work presents an innovative application of carbon dots (Cdots) nanoparticles as sensing layer for relative humidity detection. The developed sensor is based on interdigitated capacitive electrodes screen printed on a flexible transparent polyethylene terephthalate (PET) film. Cdots are deposited on top of these electrodes. An exhaustive characterization of the nanoparticles has been conducted along with the fabrication of the sensor structure. The accompanied experiments give all the sensibility to the Cdots, showing its dependence with temperature and exciting frequency. To the best of our knowledge, this work paves the path to the use of these kind of nanoparticles in printed flexible capacitive sensors aimed to be employed in the continuously expanding Internet of Things ecosystem.

## 1. Introduction

The increasing demand in sensors and actuators in recent decades has motivated the research of new sensing nanomaterials, which fulfil the desired requirements such as cost-effective production, high sensitivity, and high selectivity to the parameter of interest, among other characteristics [1]. Furthermore, though the sensing material improvement is of principal interest not least is the selection of the substrate that supports it. This selection determines the integration of the sensor with the environment where the magnitude to measure must be acquired. Ideally, the inclusion of sensor systems must be transparent to the controlled processes and above all, it should not influence the design of the process or system to be controlled.

In this context, the development of relative humidity (RH) sensors attracted considerable attention given their importance for various processes in the food industry and industrial processes [2,3,4,5]. In particular, the interdigitated electrode (IDE) has been extensively investigated to detect capacitive humidity sensors [6,7,8]. Many materials have been used to manufacture flexible relative humidity sensors in IDE systems, such as carbon nanotubes (CNTs) [9], metal and organic frameworks (MOFs) [10], and different polymers or oxides [11,12,13].

In this sense, nanomaterials (metal nanoparticles, semiconductor quantum dots, carbon dots, nanotubes, and nanocrystals) can be used as novel sensing elements and they allow us to build devices much smaller than before. There are many examples in the literature where this kind of materials are employed for different sensing applications. For example, CNTs and graphene derivates have been utilized for gas [14,15,16], temperature [17,18], and relative humidity (RH) sensing [12,19]. They offer high sensitivity and flexibility to the sensor but they lack of selectivity: they response to almost everything [20]. Other widely used nanomaterials are metal nanoparticles, which have been used for not only electrodes [21] but also together with CNTs to tune their selectivity [22,23]. Recently, new nanoparticles called carbon dots have emerged and attracted growing interest in chemistry, physics, and materials science. Carbon dots (Cdots) exhibit excellent properties, which include aqueous solubility, good electronic conductivity, low toxicity, and biocompatibility [24]. They can be synthesized using a simple, cost-effective, and environmentally friendly method at large scale. Although these Cdots-based materials hold great promise in bioimaging, drug delivery, sensors, photocatalysis, and optoelectronics [25], much work is still necessary to explore the full potentials of these nanomaterials in the development of advanced smart sensors, a new avenue for creating simple, selective, and non-invasive real-time analysis, as they can satisfy the growing demand for rapid and cost-effective quantitation [26].

In this work, we demonstrate the potential use of Cdots as sensing layer for capacitive RH and temperature sensors, contrary to other works where the use of Cdots is limited to resistive sensors [27,28,29]. The designed sensors are manufactured on a flexible substrate by means of printed electronics (PE), which allows their integration in almost any device or artefact, enabling its pervasiveness (understood as the ability to be integrated into any element of the environment), and a desired feature for the well-known Internet of Things (IoT) ecosystem. Indeed, thanks to PE, the sensors fulfil most of the desired features of the IoT paradigm, where each physical entity can be connected to its environment and share parameters of its surroundings and self-status, and therefore, they will contain more than one sensor. For these huge amount of needed devices (it is expected by 2020 to have more than 75 billion connected things [1,30]), it is mandatory to achieve cost-effective and large-scale manufacturing. Moreover, other desirable features are conformability (for adapting the electronics to the surfaces shapes) and environmental friendliness (for the reduction of generated wastes during the whole product life). All these features can be achieved by using PE [31,32]. The sensor described in this work corresponds to such devices incorporating a novel sensing element: a Cdots-based layer. It is the first time that their electrical properties towards environmental conditions have been explored to the best of our knowledge. We have also performed such analysis over frequency in order to gain a better insight of its response and try to exploit it better as in previous works [9,33].

The exhaustive presentation of the work introduced in this manuscript is structured in the following sections. Section 2 presents all the materials and methods used for developing the RH sensor: from the tools and instruments for the synthesis of the carbon dots to the fabrication and deposition technologies for the interdigitated electrode (IDE) capacitive sensor. The document continues in Section 3 showing the results obtained from de devices under test (DUT), describing the conducted experiments. A detailed discussion of these results shows the skills of these RH sensors indicating its dependence and behavior with temperature changes. Finally, Section 4 contains the conclusion of this work where its strengths are remarked.

## 2. Materials and Methods

### 2.1. Carbon Dots Synthesis

#### 2.1.1. Synthesis of Carbon Dots

The Cdots were prepared according to the procedure described by Salinas-Castillo et al. [34,35]. Rapid procedure with a dissolved citric acid and polyethyleneimine (PEI) in water, then, it was placed in microwave oven and heated at 180 °C for 5 min at a power of 850 W. The solution was dialyzed using spectra7pro dialysis membrane 1 KDa cut-off. The solution was characterized by several techniques and obtained results agree with previous works.

#### 2.1.2. Instrumentation

Microwave Milestone MicroSYNTH (Milestone Srl, Sorisole (BG), Italy) was used for synthesis. XRD was carried out at the Center of Scientific Instrumentation (University of Granada, Granada, Spain) on an EA 1108 model Fisons-Carlo Erba analyzer (Egelsbach, Germany). High-resolution transmission electron microscopy (HR-TEM) images were obtained from a FEI TITAN G2 60–300 field-emission instrument, equipped with a HAADF detector, the samples were prepared at room temperature in air by the deposition of a drop of aqueous solution of CNDs on a commercial 400 μm mesh carbon Cu-grid.

Fourier transform infrared (FTIR) spectra were obtained using a PerkinElmer FT-IR Spectrum Two spectrometer (PerkinElmer, Waltham, MA, USA). The X-ray diffraction (XRD) was carried out at the Centre of Scientific Instrumentation (University of Granada) on a Fisons-Carlo Erba analyzer model EA 1108. X-ray photoelectron spectroscopy (XPS) analyses were carried out at the Centre of Scientific Instrumentation (University of Granada), with a Kratos Axis Ultra-DLD (Kratos Analytical Ltd., Manchester, UK).

### 2.2. Sensor Fabrication

The sensors were fabricated on the transparent (polyeth)ylene ter(ephthalate) (PET) film with 125 µm. First, the interdigitated electrodes (IDEs) were screen printed with manual screen printer Nino (Coruna Printed Electronics GmbH, Bettwil, Switzerland) using a silver conductive paste (1010 from Loctite, Düsseldorf, Germany). The paste consists of 68 ± 2% silver particles. The screen mesh density was 165 T/cm. After depositing the electrodes, the samples were dried in an oven (Memmbert UF55, Memmert GmbH + Co.KG, Schwabach, Germany) at 60 °C for 60 min.

**Figure 1:**
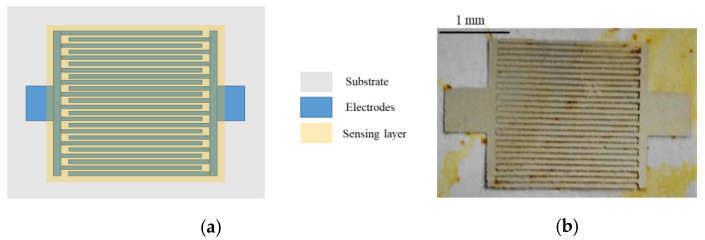
Schematic of the sensor (**a**) and real picture (**b**).

The NP were dispersed in ethanol and left in ultrasonic bath for 1 h. After that, they were drop-casted onto the electrodes and dried in a hotplate at 60 °C for 10 min. Figure 1 depicts a schematic of the sensor and a real picture of the device. The IDE structure is composed by 20 fingers per electrode of 2 mm length and 200 µm width and spacing among consecutive fingers. The resulting thickness of the electrodes after the deposition of the CDots is 4.1 ± 0.6 µm. The darker yellowish spots are accumulation of Cdots.

### 2.3. Characterization

The capacitance measurements were obtained using the E4990A impedance analyzer (from Keysight Technologies, Santa Rosa, CA, USA) with an impedance probe kit (42941A). The excitation voltage applied was V_DC_ = 0 and V_AC_ = 500 mV in all measurements and the frequency ranged from 1 kHz to 10 MHz. A calibration was performed to compensate the parasitic elements, as the one described in [33]. The sensor was placed in a climatic chamber (VCL4006 from Vötsch Industrietechnik GmbH, Balingen, Germany) with the temperature and humidity control.

For the RH sensing, the moisture content was ramped up in 10% steps and held for 1 h to ensure a stable value in the whole chamber volume. A similar approach was followed for the temperature sensing with 5 °C steps for 30 min. The measurements were automated with the use of LabVIEW 2016. The thickness of the electrodes was measured with a Dektak XT™ Stimulus Surface Profiling System (Bruker Corporation, Conventry, UK).

A custom bending setup as the one employed in [36] was used for the bending tests. The bending speed and minimum bending diameter were kept constant at 10 mm/s and 1 mm, respectively.

## 3. Results and Discussion

In this section, we first show the structural characterization of the Cdots followed by the electrical characterization of the fabricated device with respect to the moisture content at a fixed temperature. Later, we analyze the effect of temperature at different RH values.

### 3.1. Characterization of Carbon Dots

The size, morphology and structure of the Cdots were studied by high-resolution HR-TEM, EDX, XPS, FTIR, and XRD.

**Figure 2:**
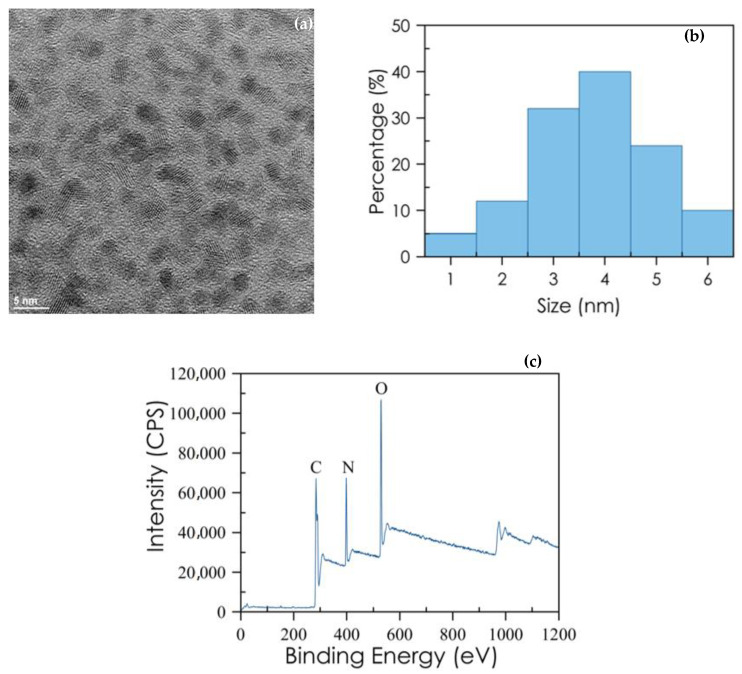
Characterization of the CDs. (**a**) HR-TEM image; (**b**) size distribution for CDs; (**c**) XPS spectra: C1s peak: 284 eV; N1s peak: 398 eV and O1s peak: 530.

The HR-TEM image of Cdots shows that the Cdots are spherical with a low degree of the agglomeration. The particle size distribution histogram is presented in Figure 2a. The size of CDs is distributed in narrow range of 1–6 nm with an average particle size of 4 nm, see Figure 2b. Elemental analyses were performed by EDX to disclose the composition of Cdots, showing that C, N, and O atoms are present on the composition of Cdots.

The FTIR spectrum of Cdots showed typical bands (COOH of 3440 cm^−1^, NH of 1585 cm^−1^, CH of 2950 and 2820 cm^−1^, CN of 1122 cm^−1^). Furthermore, the elemental analysis of the composition of the Cdots was performed by XPS surface analysis (Figure 2c). As expected, the obtained data for the elemental composition of Cdots indicate the presence of a carbon peak at about 284 eV, an oxygen peak at about 530 eV and a nitrogen peak at about 398 eV. Additionally, the atomic quantification shows 69.37% C1s, 16.62% O1s and 14.02% N1s atoms. XRD pattern had a broad diffraction peak at 2Φ = 20.5°, suggesting an amorphous material.

### 3.2. Electrical Response to RH

Before depositing the sensing layer, we measured a capacitive value of about 4 pF at 100 kHz at room conditions. We also characterized the silver electrodes on PET with the same RH swept and there was virtually no change in the capacitance over the frequency range selected (less than 3% in the whole range) [37]. Therefore, any variation in the response with respect to moisture content can be attributed to a change in the sensing layer.

**Figure 3:**
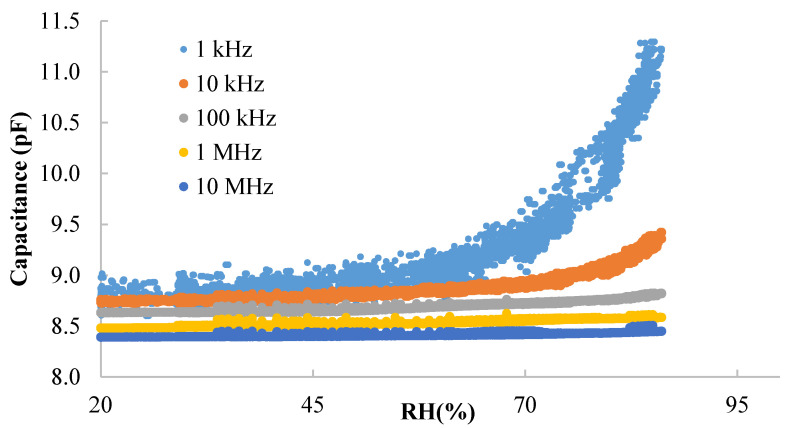
Capacitance vs. RH at different frequencies and 40 °C.

**Figure 4:**
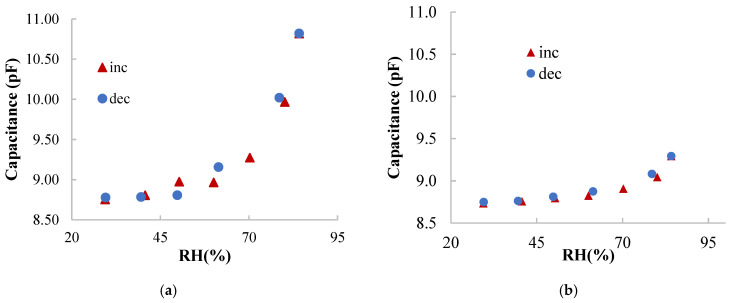
Calibration curves increasing and decreasing RH at 1 kHz (**a**) and 10 kHz (**b**). In both graphs, it is shown the responses when RH is increasing (inc) and decreasing (dec).

Figure 3 present the response of the DUT (device under test) towards RH at different frequencies. The DUT shows a capacitive behavior with about 8.8 pF measured at 100 kHz and room conditions. The highest response to RH is found at the lowest frequency. Above 100 kHz the capacitance changes less than 100 fF. Therefore, we have analyzed the response at 1 kHz and 10 kHz (see Figure 4). In terms of absolute response, the capacitance varies 2 pF at 1 kHz and 0.6 pF at 10 kHz from 20% to 85% RH. In terms of linearity, the response at 10 kHz shows a coefficient of linear regression above 0.84 while the fitting curve at 1 kHz has a coefficient of linear regression below 0.73. In fact, a better approach is to linearize the response by RH sections, defining two different curves according to the RH level (see Table 1). With respect to the hysteresis, the maximum error between the data recorded increasing and decreasing RH is about 200 fF at 1 kHz and about 50 fF at 10 kHz. In both cases, the maximum error occurs at 60% RH.

Looking at the calibration curves, they could be better fitted by two lines, one covering from 20% RH to 55% RH and the other fitting line for higher RH values, see Table 1. In fact, the sensitivity above 55% RH is one order of magnitude higher than at low RH values. In the case of the curves for higher RH values, the sensitivity is about 70 fF/% RH and 17 fF/% RH at 1 kHz and 10 kHz, respectively. It should be noted that the linear coefficient of these curves is between 0.90 and 0.95, depending on the RH level and frequency of operation. Ideally, such coefficients should be higher than 0.99. Nevertheless, although linearity is desirable for sensors, it is not crucial for their use. What is important is the fact that the sensors can discriminate RH values in the whole analyzed range.

The possible response mechanism of the synthesized Cdots can be explained with the properties of the characteristics of the surface of the nanoparticles. Previously, the polymer PEI has been used in the functionalization of graphene, introducing an electronic doping for example in carbon nanotubes [38]. PEI is known for its high hydrophilicity, which is why it has been used in humidity sensors. In said polymer, water molecules are adsorbed on PEI due to the basic nature of PEI’s hydrogen bond and PEI amines partially protonate by donating protons [39,40,41].

Regarding the bending stress, we have tested the sensors over 100 bending cycles at 1 mm length and we found a variation in the capacitance below 0.5%. The sensors were tested 6 months after its fabrication and first characterization tests, and they exhibit a variation of only 2%.

Despite the sensitivity found for this Cdots-based sensor is in the range of few fF/% RH, other printed capacitive RH sensors, which used different sensing layers, exhibited sensitivities in the same range [21,42]. For example, Molina-Lopez described a inkjet printed capacitive sensor with cellulose acetate butyrate (CAB) as sensing material with a sensitivity of 2.36 fF/% RH [42]. Moreover, in this work, we employed screen printing for the IDE definition, which provides a worse spatial resolution than other printing techniques such as inkjet printing. This poorer resolution directly affects the sensitivity because the fingers of the IDE structure cannot be defined as closer as with other technologies. However, screen printing results in a faster process, being closer to large-scale manufacturing.

### 3.3. Response to Temperature

**Figure 5:**
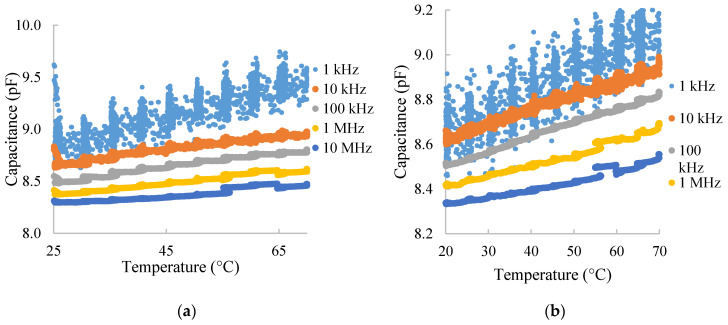
Capacitance vs. temperature at different frequencies and (**a**) 60% RH and (**b**) 40% RH.

The most interfering factor in humidity sensors is temperature. In this regard, we have analyzed its influence in our DUT, varying the temperature from 20 to 70 °C. Notice that the sensors could not be used upon 115 °C because of the glass transition point of the employed substrate. Figure 5 shows the capacitance over temperature for different frequencies at 60% RH (Figure 5a) and 40% RH (Figure 5b). It can be seen how there is a significant thermal drift in both cases, although it is slightly lower at higher RH values.

Finally, we have calculated the sensitivity to RH and the thermal drift of the sensor at the different frequencies, S_RH_ and S_T_ (f), respectively using Equations (1) and (2).

(1) $$S_{\mathrm{RH}}\;=\;\frac{\partial C_{T=\mathrm{cte}}\;\left(\mathrm{RH}\right)}{\partial \;\mathrm{RH}}.\;$$

(2) $$S_{T}\;=\;\frac{\partial C_{\mathrm{RH}=\mathrm{cte}}\;\left(T\right)}{\partial \;\mathrm{RH}}.\;$$

**Figure 6:**
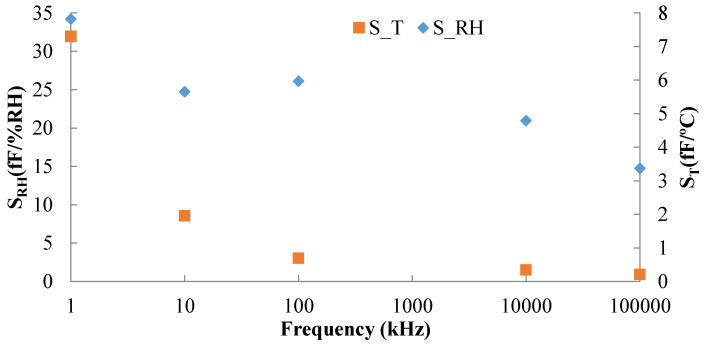
Sensitivity to RH (at 40 °C) and thermal (at 60% RH) sensitivity over frequency.

Figure 6 presents the calculated sensitivities. The dependency with respect RH is 4 times higher at 1 kHz and 1.5 times at 10 kHz than the one to temperature whereas at higher frequencies there is almost no dependency towards RH but the thermal drift cannot be neglected (even at 10 MHz the variation is more than 3 fF/°C). In fact, RH sensors are commonly affected by temperature [43], requiring thermal compensation [44,45,46]. This could be exploited as self-thermal compensated RH sensor by measuring at higher frequencies (above 1 MHz) the temperature value and using it to compensate the RH value obtained at lower frequencies (below 100 kHz).

## 4. Conclusions

Carbon dots materials have been studied for sensing devices implementations in IDE for their use for humidity sensors. Different to previous developed Cdots sensors for moisture content detection, our sensors present a capacitive response, which results in a lower power consumption and faster response in comparison with resistive behavior [47]. Furthermore, the sensors are fabricated by printing techniques on a flexible substrate, providing more interesting features, such as pervasiveness, and light-weight and cost-effective devices. Cdots can be used as sensing layer in this kind of devices, although an exhaustive characterization on frequency is mandatory.

The fabricated devices show a capacitive behavior with about 8.7 pF measured at 100 kHz and room conditions. The highest response towards RH is found at the lowest frequency. Above 100 kHz the capacitance changes less than 100 fF for the analyzed RH range. In terms of absolute response, the capacitance varies 2 pF at 1 kHz and 0.6 pF at 10 kHz from 20% to 90% RH.

Moreover, from this study, a method of temperature compensation could be used without tending an extra temperature sensor: self-compensated humidity sensor. To achieve that, the temperature will be measured at frequencies above 1 MHz, and this reading of the temperature will be employed to compensate the RH value obtained at frequencies below 100 kHz.

## Figures and Tables

**Table 1:** Calibration curves extracted from Figure 4.

Freq.	RH < 55%	RH > 55%
1 kHz	C(pF) = 0.0047·RH(%) + 8.6162 R^2^ = 0.9579	C(pF) = 0.0696·RH(%) + 4.9399 R^2^ = 0.9050
10 kHz	C(pF) = 0.0028·RH(%) + 8.6542 R^2^ = 0.9468	C(pF) = 0.0169·RH(%) + 7.7843 R^2^ = 0.8995
